# Structure of an *ex vivoDrosophila* TOM complex determined by single-particle cryoEM

**DOI:** 10.1107/S2052252524011011

**Published:** 2025-01-01

**Authors:** Agalya Periasamy, Pamela Ornelas, Thomas Bausewein, Naomi Mitchell, Jiamin Zhao, Leonie M. Quinn, Werner Kuehlbrandt, Jacqueline M. Gulbis

**Affiliations:** a Structural Biology Division The Walter and Eliza Hall Institute of Medical Research Parkville Victoria3052 Australia; b Department of Medical Biology University of Melbourne Parkville Victoria3052 Australia; c Department of Structural Biology Max Planck Institute of Biophysics Max-von-Laue-Strasse 3 60438Frankfurt am Main Germany; d Department of Cancer Biology and Therapeutics, John Curtin School of Medical Research Australian National University (ANU) Canberra ACT Australia; Chinese Academy of Sciences, China

**Keywords:** mitochondrial translocases, single-particle cryoEM, TOM complex, *Drosophila melanogaster*, membrane proteins, Tom40, macromolecular machines

## Abstract

Most mitochondrial precursor proteins are encoded in the cell nucleus and synthesized on cytoplasmic ribosomes. The translocase of the outer membrane (TOM) is the main protein-import pore of mitochondria, recognizing nascent precursors of mitochondrially targeted proteins and transferring them across the outer membrane. A 3.3 Å resolution map and molecular model of a TOM complex from *Drosophila melanogaster*, obtained by single-particle electron cryomicroscopy, is presented. As the first reported structure of a transgenic protein expressed and purified *ex vivo* from *Drosophila*, the method provides impetus for parallel structural and genetic analyses of protein complexes linked to human pathology. The core TOM complex extracted from native membranes of the *D. melanogaster* retina contains transgenic Tom40 co-assembled with four endogenous TOM components: Tom22, Tom5, Tom6 and Tom7. The *Drosophila* TOM structure presented here shows that the human and *Drosophila* TOM are very similar, with small conformational changes at two subunit interfaces attributable to variation in lipid-binding residues. The new structure provides an opportunity to pinpoint general features that differentiate the TOM structures of higher and unicellular eukaryotes. While the quaternary fold of the assembly is retained, local nuances of structural elements implicated in precursor import are indicative of subtle evolutionary change.

## Abstract 2

A 3.3 Å resolution map and structural model of the TOM complex from *Drosophila melanogaster* were determined by single-particle cryoEM. The complex was extracted from native membranes of the fly retina following targeted expression of a Tom40 transgene.

## 1. Introduction

Mitochondria have endosymbiotic origins, and the transfer of essential proteobacterial genes from mitochondria to the host nucleus necessitated the co-evolution of molecular machinery to import nuclear-encoded proteins to the organelle (Zimorski *et al.*, 2014 ▸). A suite of mitochondrial protein translocases work in concert to import different classes of precursor proteins. Mitochondria have specialized translocases in the inner membrane (TIM22 and TIM23), a redox shuttle (MIA/ERV), precursor-transfer (small TIM) components in the intermembrane space, and a sorting and assembly machinery (SAM) that folds and inserts β-barrels in the outer mitochondrial membrane (Neupert & Herrmann, 2007 ▸). The translocase of the outer membrane (TOM) is a general import pore that selectively imports nuclear-encoded mitochondrially targeted proteins and facilitates their transfer to other translocases.

The translocase function of TOM has been widely investigated over more than two decades. Well resolved structures of human and fungal TOM core complexes became tractable following a revolution in electron cryomicroscopy (cryoEM; Bausewein *et al.*, 2017 ▸; Araiso *et al.*, 2019 ▸; Wang *et al.*, 2020 ▸; Tucker & Park, 2019 ▸). TOM core complexes are membrane-embedded stoichiometric complexes with two pores, each containing five subunits: Tom40, Tom22, Tom7, Tom6 and Tom5. TOM holo-complexes contain two additional subunits involved in ensuring the fidelity of precursor transfer, Tom20 and Tom70, which share tetratricopeptide-motif folds with the ATP-dependent chaperones (Abe *et al.*, 2000 ▸; Wu & Sha, 2006 ▸). During precursor import, a flexible cytoplasmic domain of Tom20 coordinates with Tom22 in recognizing amphipathic mitochondrial targeting signals (MTS) on precursors and transferring them to the pore (Yamano *et al.*, 2008 ▸). Tom70 recognizes a different class of precursor, the inner membrane solute carriers, and its large soluble domain interacts directly with the cytoplasmic chaperone Hsp90 (Young *et al.*, 2003 ▸). After entering the general import pore of TOM, precursors reach the intermembrane space through a sequence of hydrophobic and electrostatic interactions.

Its location in the outer mitochondrial membrane also positions TOM for interaction with other proteins and organelles. A critical role of TOM is in monitoring mitochondrial health and signaling the onset of mitophagy in response to changes such as an increase in reactive oxygen species or depolarization of the inner membrane (Lazarou *et al.*, 2012 ▸). A proportion of TOM complexes are localized to mitochondria-associated ER membranes (MAMs), where they perform specialized functions, including the recruitment of autophagy-related proteins (Tang *et al.*, 2019 ▸). Cellular levels of TOM are highly regulated (Morgenstern *et al.*, 2017 ▸), and perturbation of normal levels can lead to apoptosis (Periasamy *et al.*, 2022 ▸) or autophagy (Liu *et al.*, 2018 ▸). More recently, pathological mutations identified in Tom40 and Tom7 (Chen *et al.*, 2023 ▸; Garg *et al.*, 2022 ▸; Young *et al.*, 2023 ▸) indicate that TOM may play noncanonical roles in cellular stress responses.

The main component of TOM is its translocation pore, Tom40, which is closely related to the mitochondrial porins (voltage-dependent anion channels; VDACs; Pusnik *et al.*, 2009 ▸); the 19-strand β-barrel has an internal α-helix (α2) that limits the size of the pore. However, unlike the porins (Bayrhuber *et al.*, 2008 ▸), Tom40 does not lie flat in the plane of the membrane but adopts a chevron-like quaternary fold that tilts it slightly out of the membrane plane. The small TOM subunits Tom5, Tom6 and Tom7 are membrane-embedded single α-helices that adopt characteristic shapes and interface with the outer surface of Tom40, shielding regions that are exposed to the cytosol. Tom22 is significantly larger than the small TOMs; its partially membrane-embedded helix lies at the junction of two Tom40 subunits, and a short helical region extends into the intermembrane space. Its C-terminus, which is structurally disordered, is believed to be the primary contact site for TIM23 (van Wilpe *et al.*, 1999 ▸).

CryoEM structures of TOM complexes from *Homo sapiens*, *Neurospora crassa* and *Saccharomyces cerevisiae* have been reported (Wang *et al.*, 2020 ▸; Guan *et al.*, 2021 ▸; Ornelas *et al.*, 2023 ▸; Araiso *et al.*, 2019 ▸). The *Drosophila melanogaster* TOM structure presented here shows that the human and *Drosophila* TOM are very similar, with small conformational changes at two subunit interfaces attributable to variation in the lipid-binding residues. More significantly, the *Drosophila* structure provides an opportunity to ascertain features that differentiate the TOM core structures of higher eukaryotes from their counterparts in unicellular species. Structural comparisons provide insight into how regions implicated in precursor binding and release have altered during evolution. The new structure also provides occasion to review the first identified pathological mutations of core TOM subunits in a structural context.

## 2. Materials and methods

### 2.1. *Drosophila* stocks and husbandry

The fly strains used in the experiments were obtained from the Bloomington *Drosophila* stock center and were as follows: UAS-Tom40-FLAG.HA, UAS-Myc, UAS-mito-YFP, Tubulin-GAL4/Tm6B and GMR-GAL4 (Supplementary Table S1). All crosses were cultured at 25°C. For large-scale culturing for cryoEM, crosses were mass-harvested in 6 oz polypropylene bottles (Genesee Scientific, catalogue No. 32-130). Recombinant GMR-GAL4, UAS-Tom40-FLAG-HA line was crossed to the UAS-Myc transgene to generate progeny expressing both Tom40_FLAG-HA and Myc specifically in the eye. Adult progeny were collected 2–3 days after eclosure and stored at −80°C in batches of 10 ml each (about 2000 flies in each tube).

### 2.2. Preparation of total membranes

Approximately 20 000 flies were used to purify sufficient complex for cryoEM experiments. To enrich for eye tissue expressing Tom40-FLAG-HA, fly heads were separated from bodies using a method adapted from Hackmann *et al.* (2015 ▸). Fly heads were isolated by briefly vortexing vitrified flies with 3.5 mm glass beads (BioSpec) pre-cooled in liquid nitrogen and fractionated using four stacked pre-chilled stainless-steel sieves of decreasing pore diameter (Impact Test Equipment Ltd, UK; mesh sizes 710, 500, 355 and 250 µm) with vigorous manual agitation. Fly heads collected in the 500 µm sieve were collected into a microcentrifuge tube using a funnel and were homogenized in 50 m*M* Tris–HCl pH 7.4, 150 m*M* NaCl supplemented with DNAse I (Merck–Millipore) and protease-inhibitor cocktail (Roche) using a handheld plastic pestle. The homogenized tissue was transferred to a Dounce tissue grinder using a pipette and approximately five strokes were performed with a gentle twisting motion using a loose pestle to complete the homogenization process. Crude lysates were filtered using a 100 µm filter unit to remove debris and were ultracentrifuged at 100 000*g* for 1 h at 4°C. The pellet containing the cellular membrane fraction was retained. Typically, 100 ml of flies (equating to approximately 20 000 flies) yielded 1 g of total membranes.

### 2.3. Purification of the TOM complex

Membrane pellets were resuspended in 50 m*M* Tris–HCl pH 7.4, 150 m*M* NaCl supplemented with either 1%(*w*/*v*) high-purity, low-turbidity (<2.5 NTU) water-soluble digitonin (Carbosynth, catalogue No. D-3200) or a premade mixture of 1% *n*-tetradecyl-β-d-maltopyranoside (Anatrace, catalogue No. T315) and 0.2% cholesteryl hemisuccinate (Anatrace, catalogue No. CH210). The resuspended membranes were incubated at 4°C for 90 min. The samples were centrifuged at 20 000*g* for 30 min at 4°C to remove nonsolubilized material. The filtered supernatants were applied to 1 ml M2 FLAG antibody-conjugated beads (ANTI-FLAG M2 Affinity Gel; Sigma–Aldrich, catalogue No. A2220) pre-equilibrated in 50 m*M* Tris–HCl pH 7.4, 150 m*M* NaCl containing either 0.05% digitonin or 0.005% *n*-tetradecyl-β-d-maltopyranoside plus 0.001% cholesteryl hemisuccinate (wash buffer). The beads were washed extensively in place with wash buffer, followed by five column volumes of wash buffer containing 1 *M* urea, and again with a similar volume of wash buffer containing 500 m*M* NaCl, and re-equilibrated in wash buffer. Protein reconstitution into non-ionic amphipol (NAPol; Anatrace, catalogue No. NAP) was performed on-column by incubating the beads with 0.2% NAPol in wash buffer at 4°C for 60 min. After exchange, the column was washed extensively with buffer (50 m*M* Tris–HCl pH 7.4, 150 m*M* NaCl) to remove any remaining detergent prior to elution in buffer containing 0.5 mg ml^−1^ FLAG peptide (DYKDDDDK; Sigma–Aldrich, catalogue No. F3290). After a second elution step, the eluates were pooled and concentrated using a 100 kDa Amicon centrifugal filter (Merck–Millipore).

### 2.4. Sample inspection by negative-stain microscopy

For quality inspection, 3 µl of sample was applied onto carbon-coated copper grids (400 mesh, SPI Supplies) and stained with 3 µl 2% uranyl formate. Data were collected using a Tecnai Spirit Biotwin electron microscope at 120 kV (FEI) fitted with a CCD camera (Gatan). 48 micrographs of the amphipol sample were acquired with 2 s exposure at 49 000× nominal magnification and 2.26 Å pixel size. Single-particle 2D classification was performed in *cryoSPARC* v3 (Punjani *et al.*, 2017 ▸).

### 2.5. Specimen preparation for cryoEM

Graphene grids were prepared following D’Imprima *et al.* (2019 ▸). Briefly, Quantifoil R1.2/1.3 grids (Quantifoil Micro Tools) were washed in chloroform and coated with a single layer of trivial transfer graphene (ACS Materials) by flotation. The grids were heated to 150°C for 30 min to anneal the graphene layer to their surface and washed in acetone for 1 h to dissolve the protective PMMA layer. After a final rinse in 2-propanol, the grids were dried in a nitrogen stream and stored under vacuum until use.

Immediately prior to freezing, 0.5× CMC fluorinated fos-choline-8 (Anatrace) was added to the sample and 3 µl of the final mixture was applied onto the copper side of each glow-discharged graphene-coated grid. The grids were blotted for 3 s at 100% humidity at 4°C and flash-frozen in a Vitrobot Mark IV (FEI). Four data sets were recorded, amounting to 25 017 micrographs, of which 11 990 were recorded at a 28° stage tilt. All micrographs were captured in a Titan Krios electron microscope at 300 kV (ThermoFisher Scientific) using a Gatan K3 camera in counting mode and an energy filter (Gatan BioQuantum). Dose-fractionated movies were acquired with 3.5 s exposure at a 105 000× nominal magnification, resulting in a pixel size of 0.83 Å. The total accumulated dose was 55 e^−^ A^−2^. The image defocus was in the range of −1.4 to −2.4 µm.

### 2.6. Single-particle analysis

Patch-motion correction and patch CTF estimation were performed in *cryoSPARC* v3. After manual curation of the data set, particles were blob-picked from 22 721 micrographs; the best particles were then used to train a *Topaz* (Bepler *et al.*, 2019 ▸) model within *cryoSPARC*, which resulted in 2.2 million picks across the entire data set. After 2× binned extraction, the particles were 2D classified in order to discard artifacts, and a set of 914 780 best particle images was further separated into six *ab initio* reconstructions. The top four classes and their particles were subjected to heterogeneous refinement without imposed symmetry. A subset of 480 080 particles were re-extracted in their original size and further separated into three classes through a second round of *ab initio* reconstruction and heterogeneous refinement. The best subset, resulting in 4.8 Å resolution, with 198 185 particles, was then refined in *cryoSPARC* v4. After an initial non-uniform refinement in *C*1, the particles were refined with imposed *C*2 symmetry, reaching a resolution of 3.49 Å. Local refinement in *C*2 improved the resolution to 3.35 Å, as assessed by the gold-standard Fourier shell correlation (FSC) = 0.143 criterion (see Supplementary Figs. S1 and S2 for details). Further map sharpening was performed using *deepEMhancer* (Sanchez-Garcia *et al.*, 2021 ▸); this map was used solely for visualization purposes.

### 2.7. Model building

A prediction model of *Drosophila* TOM was generated using *AlphaFold-Multimer* (Evans *et al.*, 2022 ▸) and fitted into the refined map using *ISOLDE* (Croll, 2018 ▸) within *UCSF ChimeraX* (Pettersen *et al.*, 2021 ▸) and *Coot* (Emsley *et al.*, 2010 ▸). Additional real-space refinement was performed in *Phenix* (Liebschner *et al.*, 2019 ▸), resulting in a resolution of 3.34 Å (Supplementary Table S2). Phosphatidylcholine lipids (PC) were fitted into five densities in the map. The final model included residues 55–344 of Tom40 and residues 80–123 of Tom22. The models of Tom5, Tom6 and Tom7 included residues 7–45, 7–48 and 9–54, respectively. The following segments were omitted due to poor density: residues N-54 of Tom40, N-76 and 124-C of Tom22, N-6 and 46-C of Tom5, N-6 and 48-C of Tom6 and N-8 of Tom7.

## 3. Results

### 3.1. Co-expression with Myc enhances the yield of TOM

**Figure 1:**
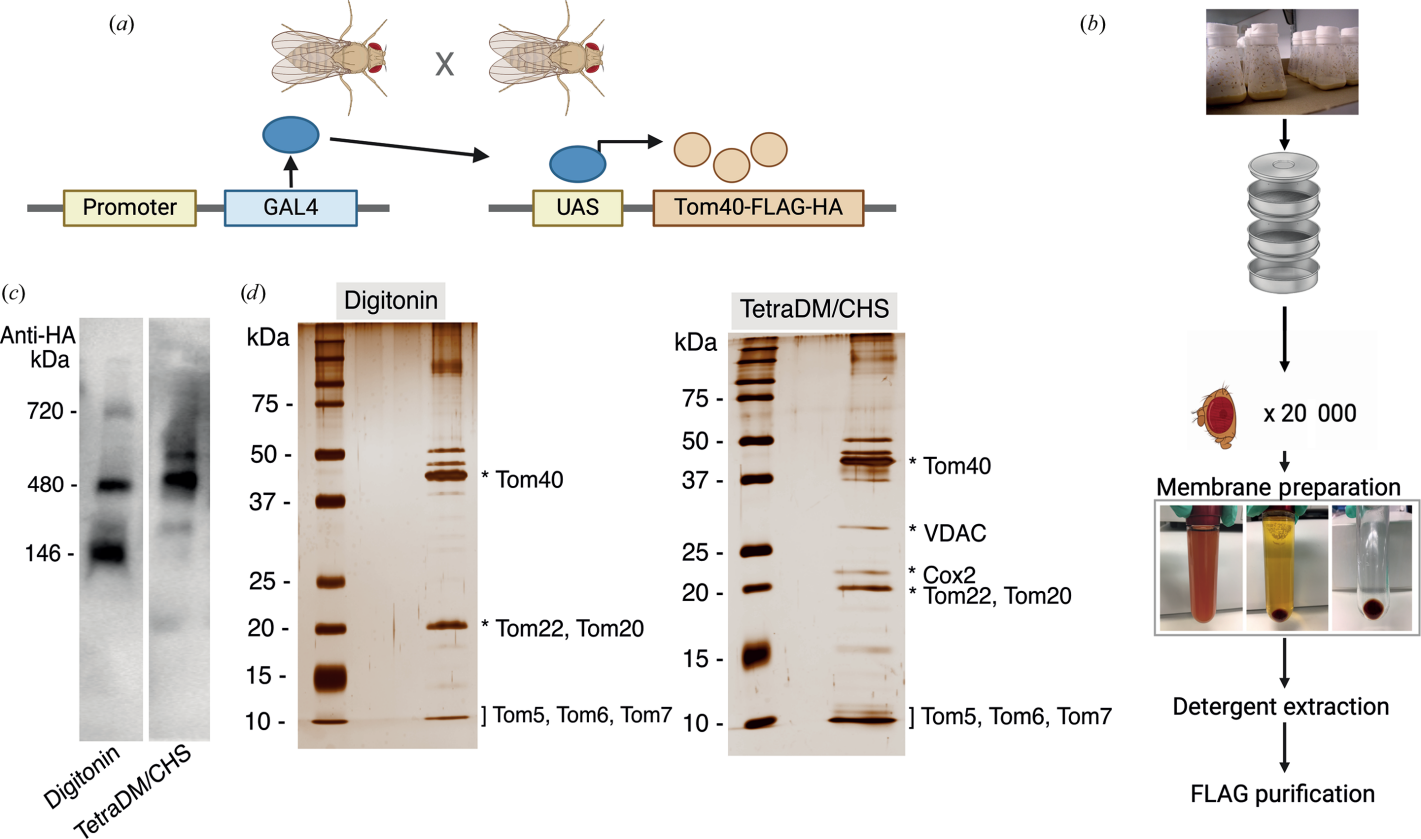
Isolation of a *Drosophila* TOM complex. (*a*) Schematic representation of the UAS–GAL4 protein-expression system in *Drosophila*. The promoter (GMR) drives ectopic expression of Tom40 in fly retina via the transcription factor GAL4 and the GAL4 response element UAS. This image was created with BioRender.com. (*b*) Workflow of protein purification. Fly heads were separated using a metal sieve stack. Fly heads collected in the 500 µm compartment were homogenized and membranes were prepared by ultracentrifugation of the lysate. Membranes were detergent-solubilized and the protein was affinity-purified using anti-FLAG resin. (*c*) Comparative BN-PAGE Western blot (anti-HA) analysis of eye membranes, expressing Tom40 (co-expressed with Myc), solubilized with either digitonin or *n*-tetradecyl-β-d-maltopyranoside (TetraDM)–cholesterol hemisuccinate (CHS). Additional bands migrating close to the 146 and 720 kDa markers indicate dissociated complexes and super-complexes, respectively. (*d*) Silver-stained SDS–PAGE gel analysis of immuno-affinity-purified TOM complex reconstituted into non-ionic amphipol. Protein identities of prominent gel bands detected by tryptic digest mass spectrometry are labeled with asterisks.

We employed *Drosophila* as an *in vivo* expression host for transgenic expression of the *Drosophila* Tom40 subunit fused to a C-terminal FLAG-HA epitope tag (Fig. 1 ▸). The bipartite UAS–GAL4 system [Fig. 1 ▸(*a*)] (Bischof *et al.*, 2007 ▸) facilitates the use of a range of promotors that target expression to specific tissues and developmental stages. Optimal Tom40 expression was achieved using a GMR promotor, which targets protein expression to the photoreceptor cells of the fly retina. Retinal neurodegeneration caused by overexpression of the transgene (Periasamy *et al.*, 2022 ▸) caused a reduction in the viable tissue available for protein expression, and hence even with this strong promotor yields were limited. To improve the yield of TOM, we exploited findings that overexpression of the oncogenic transcription factor Myc in *Drosophila* leads to a higher cellular mitochondrial content (Mitchell *et al.*, 2015 ▸). Co-expression of Myc under the GMR promotor increased transgenic Tom40 expression significantly [Supplementary Figs. S3(*a*) and S3(*b*)], and other endogenous mitochondrial proteins [Supplementary Fig. S3(*b*)], indicative of a general increase in mitochondrial content. Unexpectedly, Myc expression also completely suppressed the apoptosis of photoreceptor cells driven by expression of the Tom40 transgene, resulting in an increase in viable eye tissue [Supplementary Fig. S3(*c*)]. Overall, this strategy resulted in an enhancement of the yield of TOM for structural studies [Fig. 1 ▸(*d*)].

### 3.2. Isolation and reconstitution of *Drosophila* TOM

Blue native polyacrylamide gel electrophoresis (BN-PAGE) analysis of detergent-solubilized fly head membranes [Fig. 1 ▸(*b*)] ascertained that tagged Tom40 predominantly assembled into an ∼480 kDa band [Fig. 1 ▸(*c*)] corresponding to the TOM complex (Dekker *et al.*, 1998 ▸). Solubilization with digitonin destabilized the complex, resulting in a dissociated lower-order species that was not observed when the membranes were solubilized with an *n*-tetradecyl-β-d-maltopyranoside–cholesterol hemisuccinate combination [Fig. 1 ▸(*c*)]. The TOM complex was extracted from solubilized lysates of fly head membranes by FLAG-based affinity chromatography. A single-step on-column protocol was developed to minimize protein loss during purification. This included sequentially washing the bound complex with buffer solutions containing 0.5 *M* NaCl and 1 *M* urea. Tryptic digest mass spectrometry verified five endogenous TOM subunits that co-purified with Tom40 to be Tom22, Tom5, Tom6, Tom7 and Tom20 [Fig. 1 ▸(*d*)].

Preliminary negative-stain analysis revealed an accumulation of excess detergent during the final protein-concentration steps. This issue was resolved by an on-column reconstitution into a non-ionic amphipol (Bausewein *et al.*, 2017 ▸), resulting in a cleaner data set. Upon visual inspection and 2D classification of negative-stain micrographs of the new preparation, we found a heterogeneous range of TOM complexes varying in the number of pores (Supplementary Fig. S4). This may suggest that Tom40 or contaminating VDAC [Fig. 1 ▸(*d*)] (Künkele *et al.*, 1998 ▸) assemble into higher-order oligomers. On cryoEM grids, however, only two-pore TOM complexes were visible. This may be a consequence of dissociation into dimers and monomers during cryo-grid preparation, as noted previously (Araiso *et al.*, 2019 ▸).

### 3.3. Structure determination

Given the low sample concentrations, in-house graphene back-coated copper grids were utilized to improve the particle density for cryoEM. Unfortunately, interaction of the protein with the graphene support led to orientational bias, with the flat faces of TOM as the preferred orientations, resulting in anisotropic resolution. To compensate, a large micrograph data set was collected, with approximately half of the data collected on a tilted microscope stage.

**Figure 2:**
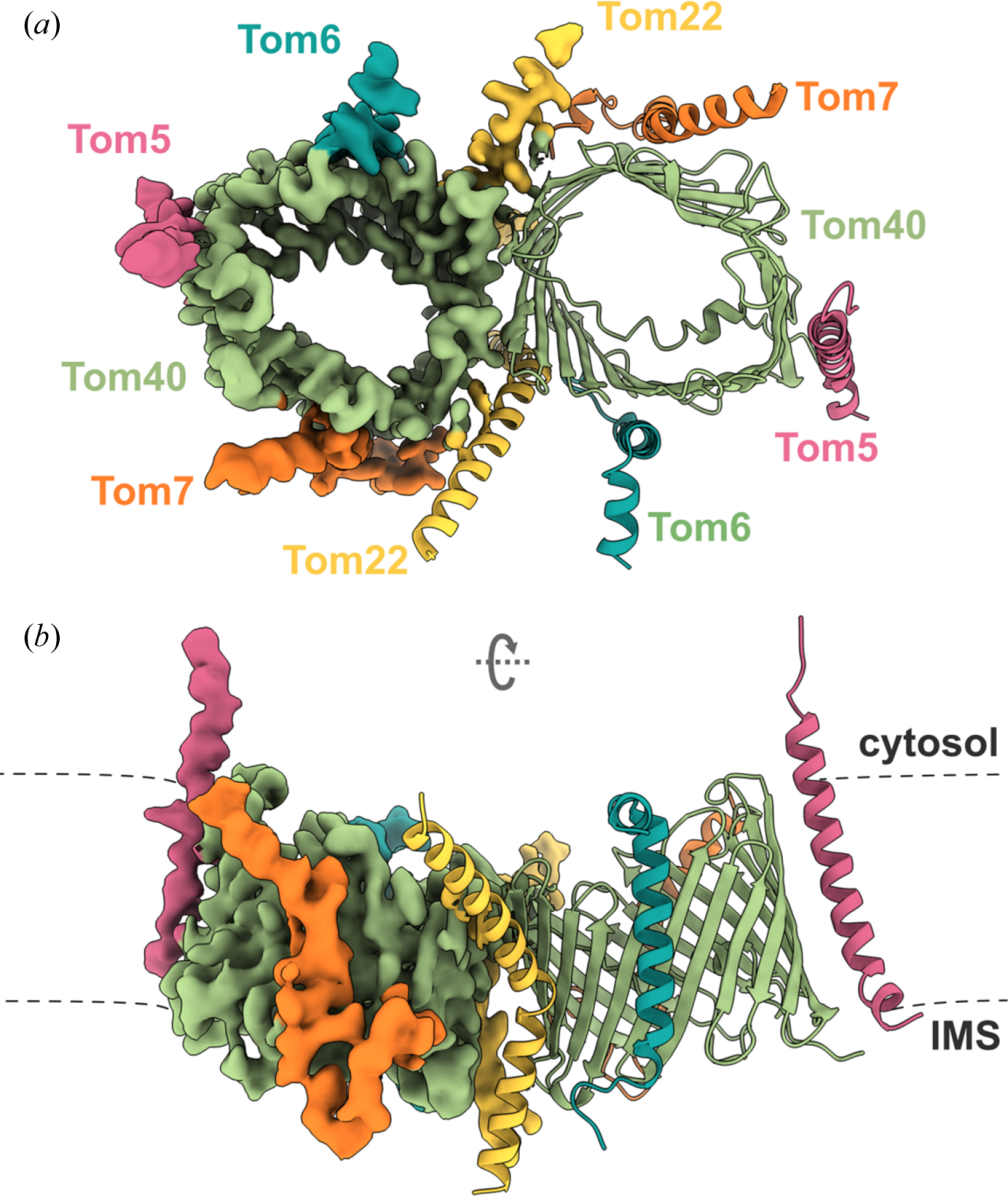
Structure of the core TOM complex of *D. melanogaster* obtained by single-particle cryoEM. A composite structure of the map and ribbon diagrams of the model of the two-pore assembly are viewed from the cytosol and from within the plane of the membrane. The subunits are colored as follows: Tom40, green; Tom22, yellow; Tom7, orange; Tom6, teal; Tom5, pink. The membrane is indicated by dashed lines.

A molecular model of the two-pore complex was built into the 3.3 Å resolution map (Fig. 2 ▸) starting from a predicted structure generated using *AlphaFold-Multimer*. The final model contains all subunits of the core complex, comprising two copies of the 19-stranded β-barrel Tom40, two copies of the precursor receptor subunit Tom22 and two copies each of Tom5, Tom6 and Tom7. The Tom40 model is missing only the disordered N-terminal domain in the intermembrane space, while Tom22 is missing its disordered N- and C-terminal domains. All three small TOM subunits (Tom5, Tom6 and Tom7) interacting with the Tom40 barrels reveal intact membrane-spanning regions.

**Figure 3:**
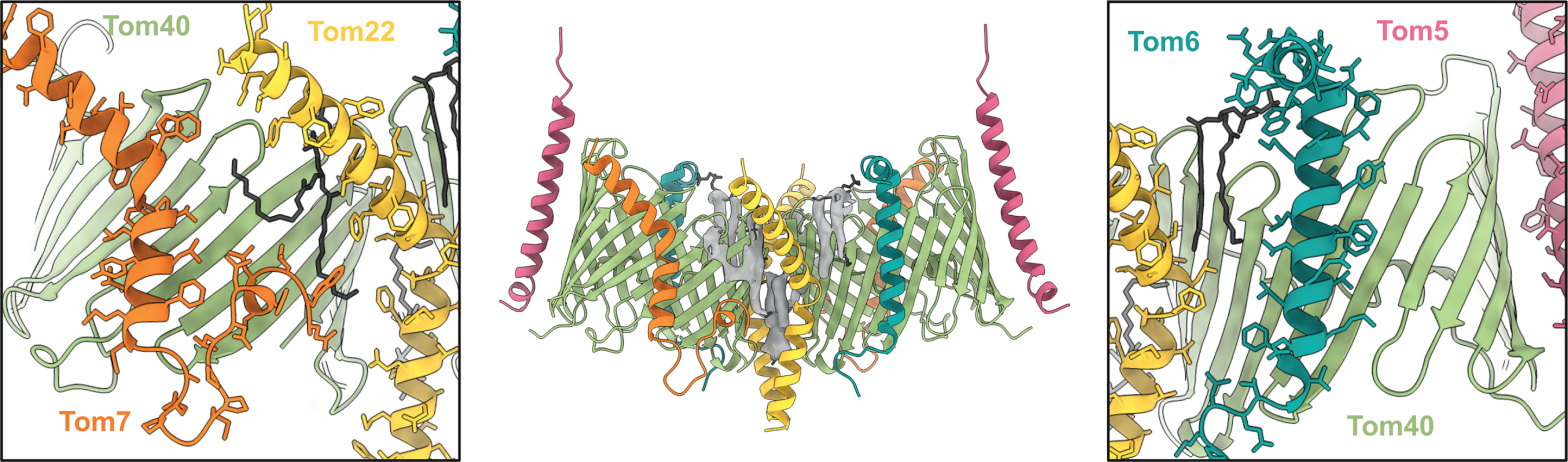
Phospholipids bound at subunit interfaces mediate subunit interactions. Density contoured at a threshold of 0.111 is shown in the center. A close-up of the interface between Tom7 and Tom22, with modeled lipid, is shown in the left inset. The right inset depicts a modeled lipid at the Tom22–Tom6 interface. Lipid molecules are shown in black.

Several bound lipids near where Tom22 and Tom40 intersect were included in the model (Fig. 3 ▸). The map resolution was insufficient to identify the lipid headgroups, and thus all were conservatively modeled as phosphatidyl choline (PC), as mass-spectrometric studies of the human TOM complex had indicated that most bound lipids were PC (Wang *et al.*, 2020 ▸).

Positive Y-shaped density for a conserved lipid-binding site between the two β-barrels has been observed in other TOM complexes (Su *et al.*, 2022 ▸; Wang *et al.*, 2020 ▸), and represents an average of two conformations related by the molecular dyad. In keeping with most other reported structures, we modeled this lipid as a single phosphatidyl choline with full occupancy (Supplementary Fig. S5).

### 3.4. Comparative analysis of core TOM structures

Our model is in close agreement with all other published cryoEM structures of TOM core complexes, especially human (*Homo sapiens*) TOM (*Hs*TOM). The common architecture of the β-barrel, Tom40, with 19-antiparallel β-strands, is conserved in *Drosophila*. The Tom40 dimer interface is formed where the first and last β-strands of each barrel, β1 and β19, meet. The two β-barrels interface directly at the cytosolic face, but their separation increases in the inner leaflet of the membrane where Tom22 subunits are interposed between the barrels. The two β-barrels are thus mutually inclined at approximately 20°, exhibiting the characteristic chevron-like TOM architecture. To highlight differences between TOMs from higher and lower eukaryotes, we compared *D. melanogaster* TOM with previously published TOM models from *Homo sapiens* (PDB entry 7ck6), *Saccharomyces cerevisiae* (PDB entry 6ucu) and *Neurospora crassa* (PDB entry 8b4i) (Supplementary Fig. S6).

#### 3.4.1. Human and *Drosophila* TOM complexes have minor differences

**Figure 4:**
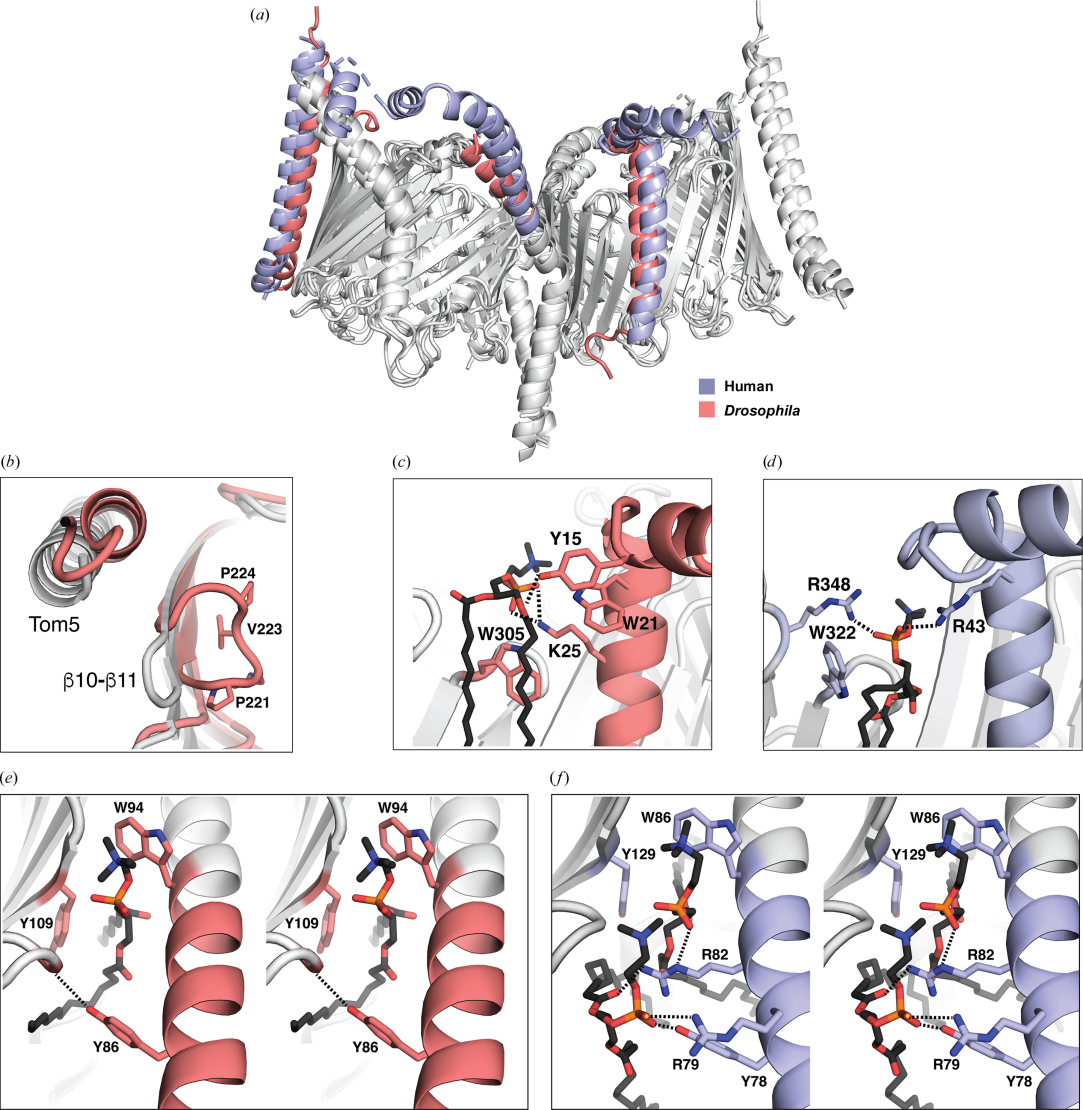
Structural differences in the core TOM structures from human and *Drosophila*. (*a*) Overlay of representative structures viewed in the plane of the membrane. To simplify, key differences are highlighted in color: *Drosophila melanogaster* TOM (PDB entry 9etm) is in salmon pink and human TOM (PDB entry 7cp9) is in lilac. (*b*) A proline-rich β10–β11 loop points into the interior of the β-barrel in *Drosophila* TOM. A lipid is modeled at the Tom6–Tom40 subunit interface of (*c*) *Drosophila* TOM and (*d*) human TOM. Stereoviews of the Tom22–Tom40 interface of (*e*) *Drosophila* and (*f*) human TOM, showing the respective effects of one or two bound lipids on the Tom40–Tom22 departure angle.

The differences between the human and *Drosophila* TOM complexes are relatively minor [Fig. 4 ▸(*a*)], as exemplified by a root-mean-square deviation (r.m.s.d.) of 1.18 Å over 236 (of 284 possible) C^α^ atoms between our Tom40 model (*Drosophila* TOM) and *Hs*TOM (PDB entry 7ck6). Unique to *Drosophila* Tom40, the proline-rich loop β10–β11 (^220^GPNVPGR^226^) points into the interior of the β-barrel. Its central valine (Val223) curves into the interior, making van der Waals interactions with side chains of the inner staves. The altered conformation may explain a small relative shift of the adjacent Tom5 α-helix in *Drosophila* TOM [Fig. 4 ▸(*b*)]. In *Neurospora* and *Saccharomyces* the cytosolic ends of the β10–β11 strands are lifted out of the plane of the membrane, whereas in human Tom40 β10–β11 is truncated to the edge of the β-sheet.

*Drosophila* Tom6 is shorter than the human equivalent, and some angular divergence between the two structures near the cytoplasmic surface of the membrane can be attributed to differences in the mode of binding of a phospholipid at the Tom40–Tom6 interface. In the *Drosophila* structure, the phospholipid is held in place by a hydrogen bond between the Tyr15 ring hydroxyl of Tom6 and an acyl moiety of the lipid, with Trp21 of Tom6 making a close approach and Trp305 of Tom40 packing against the acyl chains. The side chain of Lys25 of Tom6 interacts with the phosphate group [Fig. 4 ▸(*c*)]. By contrast, in human TOM only one aromatic residue (Trp322 of Tom40) interacts with an acyl group, while two basic residues (Arg43 of Tom6 and Arg348 of Tom40; the latter possibly water-mediated) pair with phosphate O atoms. Both sequences favor lipid binding, but in the human structure the lipid is slightly closer to Tom6 [Fig. 4 ▸(*d*)]. In the *Drosophila* structure, the lipid headgroup protrudes further out of the membrane than its counterpart in human TOM.

A subtle conformational change of similar ilk is also present at the cytoplasmic face, this time where Tom40 meets Tom22. The angular separation between Tom22 and Tom40 alters in correspondence with the number of lipids accommodated at the subunit interface: one lipid in *Drosophila* [Fig. 4 ▸(*e*)] and two in human [Fig. 4 ▸(*f*)]. The human TOM structures with PDB codes 7cp9 and 7ck6 both exhibit the wider angle, although lipids were not modeled in the latter. In both human and *Drosophila* TOM, a completely conserved Tom40 tryptophan (Trp86 in human and Trp94 in *Drosophila*) forms the back of the lipid-binding pocket, packing against the innermost lipid, while a completely conserved tyrosine in Tom40 (Tyr129 in human and Tyr109 in *Drosophila*) stabilizes the Trp21 acyl packing. In the human structure with PDB code 7cp9, the additional Tom22 arginine residues Arg79 and Arg82 accommodate the binding of the ‘outer’ lipid. While Arg82 interacts with the acyl groups of the outer lipid and the phosphate moiety of the innermost lipid, Arg79 interacts with the phosphate of the outer lipid. In *Drosophila*, the lipid headgroup is enclosed, due to Tyr86 of Tom22 interacting with the backbone carbonyl of Pro136 of Tom40. In the human structure, the corresponding tyrosine (Tyr78) interacts with the outermost lipid, which effectively acts as a spacer between the two subunits.

Thus, in both examples sequence and conformational differences between the human and *Drosophila* structures are associated with altered lipid binding at subunit interfaces.

#### 3.4.2. Structural differences between higher and lower eukaryotes

Despite the conservation of the quaternary structure of the TOM complex, local differences between the higher eukaryotes and fungi are marked. Here, we highlight differences between the structures of the *Drosophila* and *Neurospora* (PDB entry 8b4i) TOM subunits.

**Figure 5:**
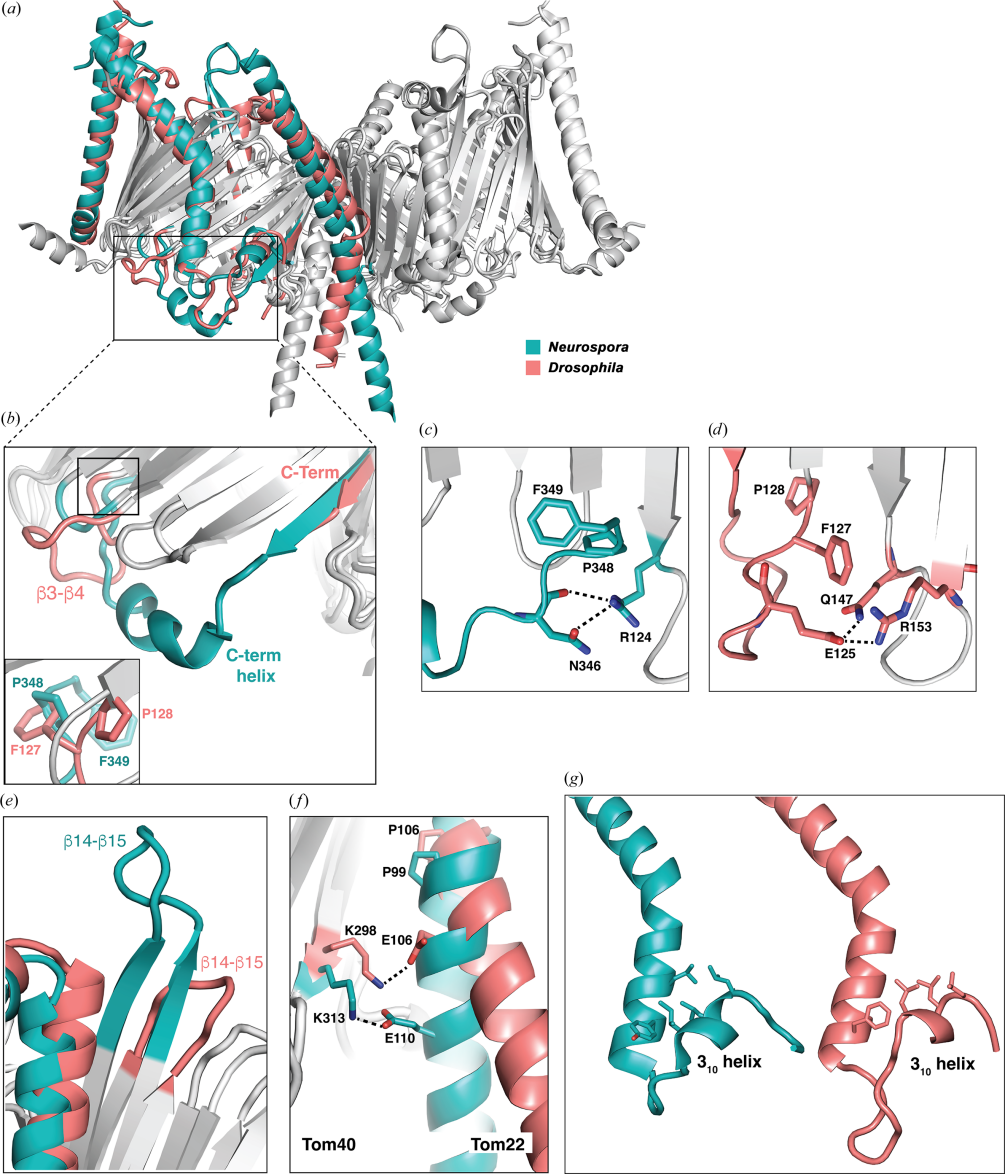
A comparison of core TOM structures from lower and higher eukaryotes. (*a*) A superposition of the *Drosophila* and *Neurospora* structures, with key differences depicted in color: *Neurospora crassa* (PDB entry 8b4i) is in teal and *Drosophila melanogaster* (PDB entry 9etm) is in salmon pink. (*b*) The C-terminal helix (α3) and loop region of *Neurospora* Tom40, interacting with a minimal β3–β4 loop (teal), is viewed in the plane of the membrane. In *Drosophila* TOM, like other higher eukaryotes, the C-terminus is at the end of strand β19 and the proximal β3–β4 loop is more extended. The inset shows a close-up of the boxed area. (*c*) The C-terminal region of *Neurospora* Tom40 viewed from the inside of the Tom40 barrel, showing a Phe–Pro motif implicated in precursor binding. (*d*) The same view of *Drosophila* Tom40 shows that a similar motif is present but derives from the extended β3–β4 loop. Both folds are stabilized by hydrogen bonding. (*e*) An overlay of the β14–β15 loop of Tom40 in the two structures, showing the longer β-strands of the fungal TOMs. (*f*) Overlay showing Tom22 in color, showing ion-pairing interactions that influence the magnitude of the kink at a conserved proline. (*g*) Side-by-side views of *Drosophila* Tom7 and *Neurospora* Tom7, revealing the disposition of branched aliphatic side chains (Leu48, Leu51 and Leu52 in *Drosophila*; Leu45, Leu48 and Leu49 in *Neurospora*) on one face of the 3_10_-helix. In *Neurospora* these favor intramolecular contacts with Tyr36 and Leu32 of Tom7, while in the *Drosophila* (and human) structures the side chains face away.

At 1.16 Å, the r.m.s.d. between C^α^ atoms of *Drosophila* Tom40 and *Neurospora* Tom40 (PDB entry 8b4i) in the conserved regions is comparable to that between the human and *Drosophila* structures (1.18 Å). Regions that deviate include the C-terminus, interstrand loops and subunit interfaces. In *Neurospora*, the helical C-terminus (α3) of Tom40 interacts with the short intermembrane space loop connecting the β3 and β4 strands [Figs. 5 ▸(*a*) and 5 ▸(*b*)]; the helix is a contact point for translocating presequences (Ornelas *et al.*, 2023 ▸). In *Saccharomyces* the C-terminal helix is present but veers away from the β3–β4 loop in the structure. Both the *Drosophila* and human Tom40 structures lack a C-terminal helix, terminating at the 19th stave of the barrel [Fig. 5 ▸(*b*)]. They compensate with a much longer β3–β4 loop, which curves in over the barrel cavity, reminiscent of a protein-recognition motif. Sequence alignments (Supplementary Fig. S7) suggest that this will be the case for all higher eukaryotes of the animal kingdom. Given the proximity, some interesting parallels can be drawn between the β3–β4 loop in *Drosophila* Tom40 and the C-terminus of *Neurospora* Tom40. Whereas in *Neurospora* a ^348^Pro-Phe^349^ motif at the C-terminus interacts with the inside of the Tom40 β-barrel, in *Drosophila* a ^127^Phe-Pro^128^ motif in the elongated β3–β4 loop occupies the same relative position, with Pro348 of the *Neurospora* C-terminus superimposing with Phe127 of the *Drosophila* β3–β4 loop [Fig. 5 ▸(*b*), inset]. In each instance, a polar residue just prior to the motif stabilizes the fold by forming hydrogen bonds; in *Neurospora* Asn346 (both its side chain and peptide carbonyl) make hydrogen bonds with Arg124, whereas in *Drosophila* they are formed by Glu125 (Gln147⋯Glu125⋯Arg153) at approximately the same relative position [Figs. 5 ▸(*c*) and 5 ▸(*d*)].

In a *Neurospora* model of Tom20, the Tom20 cytoplasmic domain is very close to a prominent structured cytosolic loop connecting strands β14 and β15 of Tom40 (Ornelas *et al.*, 2023 ▸), consistent with reported cross-linking data (Shiota *et al.*, 2015 ▸) and implying that the loop may have a role in precursor transfer. In both *Drosophila* and human TOM homologues this loop is truncated to the much shorter β-strands [Fig. 5 ▸(*e*)].

#### 3.4.3. Tom22 from higher eukaryotes has a pronounced bend

The ordered segment of Tom22 comprises a single transmembrane helix. It is bent near the center of the membrane, due to a conserved proline, and the bend is significantly more pronounced in the *Drosophila* and human TOM structures than in their fungal counterparts. The distinctive angle [Fig. 5 ▸(*a*)] as the helix protrudes into the intermembrane space results in a less than a 50% atom overlap between Tom22 in *Drosophila* and *Neurospora* TOM. Analysis suggests that the exit angle is determined by ion-pairing interactions, which differ in the two classes. The first charged residue of Tom22 C-terminal to the conserved proline is a glutamate. In *Drosophila* Tom22, the glutamate Glu110 is one turn of helix below the proline (Pro106) and is within the plane of the membrane. Ion pairing is thus obligate, due to the energetic cost of maintaining charged side chains in an apolar environment. In contrast, its counterpart in *Neurospora* (Glu106) is two helical turns below the proline (Pro99) and exists within a more polar environment nearer the mitochondrial intermembrane space. In both cases, the Tom22 glutamate forms ion-pairing interactions with a conserved basic residue in Tom40 (Tom22 Glu110⋯Tom40 Lys313 in *Drosophila*; Tom22 Glu106⋯Tom40 Lys298 in *Neurospora*) [Fig. 5 ▸(*f*)]. Within the intermembrane space, the angular difference leads to further changes. In the TOM structures from fungi, Tom22 packs close to the β15–β16 loop, stabilized by ion pairing between Tom22 Asp107 and Tom40 Arg278 [Fig. 5 ▸(*g*)], while in human and *Drosophila* Tom22 packs between the β15–β16 loop and the β17–β18 loop.

#### 3.4.4. A 3_10_-helix in Tom7 binds the aliphatic tails of membrane lipids

Tom7 has also diverged subtly in higher and lower eukaryotes. In all structures, a short 3_10_-helix near the C-terminus of Tom7 is partially submerged into the outer membrane [Fig. 3 ▸(*e*)]. The helix is stabilized at its C-terminal end by inter­actions with a conserved histidine residue of Tom40 (His97 in *Drosophila*). However, slight differences in its angular placement distinguish the TOM structures from higher and lower eukaryotes. In a pocket between the Tom40 and Tom7 sub­units, an array of leucine side chains on one face of the 3_10_-helix (Leu48, Leu51 and Leu52 in *Drosophila*) interact with lipid tails from the outer leaflet [Fig. 5 ▸(*g*)]. In *Neurospora* and *Saccharomyces*, the motif is more involved in intra­molecular contacts with the transmembrane helix. Moreover, a proline-rich ‘turret’ between the 3_10_-helix and transmembrane helix of Tom7 that protrudes into the intermembrane space (Fig. 3 ▸), giving Tom7 a saxophone-like profile in the human and *Drosophila* structures (Fig. 2 ▸), is truncated to the membrane in the fungal species. Excluding the turret region, the r.m.s.d. between Tom7 in the two species is 1.03 Å (over 33 C^α^ atoms).

## 4. Discussion

We have presented the structure of a TOM core complex purified *ex vivo* from *D. melanogaster* and described general differences between TOMs from unicellular and multicellular eukaryotes. Here, we discuss our findings and also place recently discovered disease-associated point mutations, the first to be identified in core TOM components, in the context of molecular structure.

### 4.1. Changes in the regions implicated in precursor binding

The quaternary fold is highly conserved in all TOM structures, despite only moderate sequence homology between unicellular fungi and higher eukaryotes (Supplementary Table S3). Some significant differences in detail, however, appear to reflect mechanistic nuance. Structural elements of Tom40 implicated in the capture and release stages of precursor translocation through the import pore of *Neurospora* TOM include inter-strand loops (β14–β15 and β3–β4) and the C-terminal helix (Ornelas *et al.*, 2023 ▸). These are altered in the *Drosophila* and human TOM structures, which may affect the route that precursors take to exit the TOM pores.

Structural alignments indicate that the C-terminal helix of Tom40, which is located on the intermembrane-space side of the β-barrel, is not present in higher eukaryotes. Thus, it cannot contribute to precursor binding in higher eukaryotes. However, we have identified potential structural homology between a key region of the C-terminus of *Neurospora* Tom40 and an elongated β3–β4 loop in human and *Drosophila* Tom40 that interacts with the interior of the β-barrel in a similar way and could potentially compensate for the precursor-binding site observed in *Neurospora* TOM. A caveat is that similar interactions are not observed in the *Saccharomyces* TOM structure (PDB entry 6ucu), where the C-terminal helix is less well ordered.

### 4.2. Four disease-associated TOM mutations map to the Tom40–Tom7 interface 

**Figure 6:**
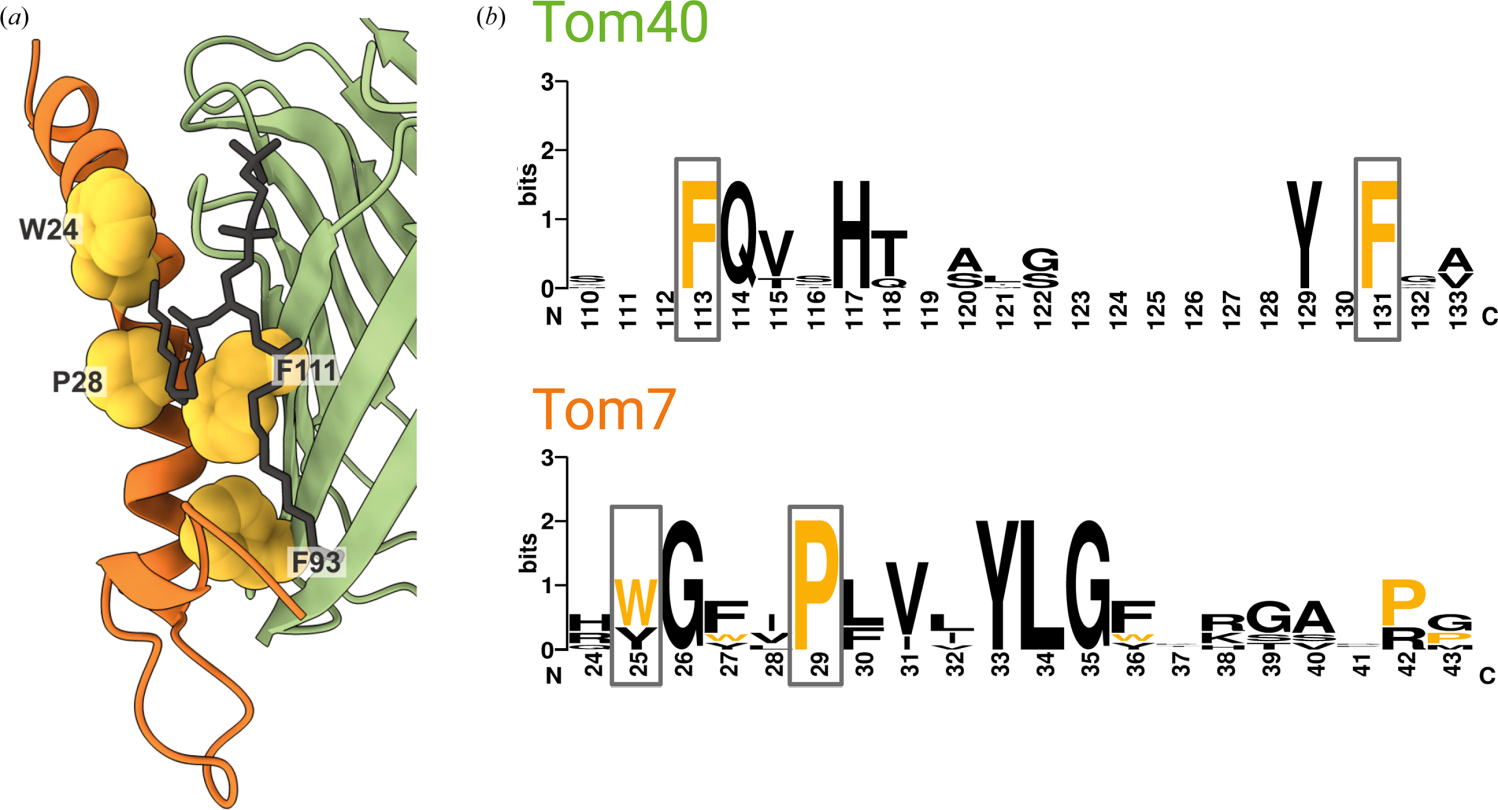
Disease-associated TOM mutations. (*a*) Cartoon representation of the *Drosophila* Tom40–Tom7 interface. Disease-associated mutations are depicted as yellow spheres. (*b*) Sequence logo (Crooks *et al.*, 2004 ▸) showing the conservation (overall height) and residue prevalence of sections of the Tom40 and Tom7 protein sequences. Residue numbering is for the human proteins. Amino-acid residues that are mutated in disease conditions are colored yellow and marked with boxes. This image was created with *WebLogo*.

Until recently, the only missense disease mutations in TOM had been identified in Tom70, a precursor receptor present only in the holo-TOM complex. Starting in 2022, two mutations of Tom7 and two of Tom40 came to light in rapid succession, illuminating an interesting pathophysiology of core TOM. All four residues are highly conserved [Figs. 6 ▸(*a*) and 6 ▸(*b*)]. A proline-to-leucine mutation of Tom7 (Pro29Leu in human; Pro28 in *Drosophila*; Garg *et al.*, 2022 ▸) in a homozygous patient (21 years of age) was linked to autosomal recessive progeria. Previous experiments have suggested that a proline-to-leucine mutation at this position could prevent efficient Tom7 targeting of mitochondria (Allen *et al.*, 2002 ▸). Structurally, the proline is part of a conserved G*xx*P motif, where the first *x* is an aromatic residue and the second is a branched aliphatic residue The two central residues of the motif interact directly with pockets on the surface of Tom40 (Supplementary Fig. S8). The very long transmembrane helix of Tom7 bends significantly, allowing an amphipathic N-terminal segment to run along the cytosolic ends of the barrel staves, making extensive contacts with β-strands 1–8. The presence of the proline supports a contiguous Tom7–Tom40 interface, which could be compromised in the disease-associated Pro→Leu variant.

The link with several diseases suggests that the Tom40–Tom7 interface has functional significance. A second human Tom7 mutation, Trp25Arg (Trp24 in *Drosophila*), again homozygous, is associated with severe developmental retardation and shortened lifespan (Young *et al.*, 2023 ▸). In the human TOM structure, the tryptophan forms part of a phospholipid-binding pocket (that also involves Tom22 and Tom40) on the cytoplasmic side of the membrane [Figs. 4 ▸(*e*) and 4 ▸(*f*)]. Thus, in the mutant, two potentially positively charged side chains of Tom7, rather than one, are in the direct vicinity of the lipid phosphate. In the *Drosophila* TOM structure the site appears very similar, but we do not observe well resolved density for an outer lipid headgroup. In the absence of the lipid-mediated contact, the cytosolic end of Tom22 is positioned closer to Tom7.

Point mutations of Tom40, Phe113Leu and Phe131Leu (Phe93 and Phe111 in *Drosophila*), have also recently been identified and have been implicated in neuroinflammation in Alzheimer’s disease (Chen *et al.*, 2023 ▸). These residues are highly conserved, as are their interactions. Both Tom40 mutations are located on the surface of the β-barrel, mapping to its interface with Tom7. While Phe113 (Phe93 in *Drosophila*) exclusively contacts Tom7, Phe131 (Phe111 in *Drosophila*) also packs against the acyl chains of a lipid bound at the interface. The Tom40–Tom7 interface thus hosts all four of the disease-linked mutations of the core complex identified to date. It has been suggested that Tom7 may assist with the dissociation of a folded Tom40–Tom5–Tom6 intermediate from SAM (Wang *et al.*, 2021 ▸). Inspection of the structure of an import-assembly complex containing the SAM β-barrel, open on one side, associated with pre-assembled Tom40, Tom5 and Tom6, reveals a minor clash of SAM with the Tom7 3_10_-helix observed in TOM structures; this is consistent with inhibition of SAM release as a possibility. However, considering that the protein-import function of TOM is essential (Kiebler *et al.*, 1990 ▸; Baker *et al.*, 1990 ▸), and that patients carrying these mutations survive to adulthood (with the exception of the Trp25Arg mutation), it seems likely that sufficient functional TOM exists in the outer mitochondrial membrane for precursor import. Alternative explanations might be that these sites are important for the interaction of TOM with other proteins or that the mutations affect non-canonical functions of TOM.

### 4.3. *Drosophila* as a model organism in structural biology

*Drosophila* has been extensively utilized to model neurodegenerative diseases, and the rationale behind our present study was to explore in principle whether protein complexes for structural analysis could be purified directly from disease models. Previously, transgenic expression analysis of G-protein coupled receptors (GPCRs) targeted to the expansive membrane stacks in the retina have been reported (Hackmann *et al.*, 2015 ▸), with a view to future structural analysis. More recently, the structure of an abundant endogenous respiratory complex extracted from native *Drosophila* has been published (Agip *et al.*, 2023 ▸). Our novel approach was ectopic expression of fusion-tagged Tom40 transgenes, using affinity chromatography of the expressed protein to purify intact TOM core complexes from fly tissue.

A key strength of the method lies in the *in vivo* correlation of structure to physiological function. In *Drosophila*, protein expression can be directed to specific tissues, cell types or developmental stages by the selection of the promotor. In our case, expression in the retina led to an unexpected discovery that Tom40 overexpression drives retinal neurodegeneration, providing insight into a strong risk factor in Alzheimer’s disease afforded by intronic ′523 *TOMM40* variants that exhibit comparable levels of increased *TOMM40* expression (Roses *et al.*, 2010 ▸). It further provided an *in vivo* system to investigate the underlying cellular mechanisms through genetic screening experiments (Periasamy *et al.*, 2022 ▸) and test hypotheses. This highlights the potential of using *Drosophila* to correlate different types of data from a single source, including structure, imaging, genetics and omics (for example lipidomics).

While immortalized cell lines are widely used for expression of mammalian proteins, their proteomes can alter over time (Pan *et al.*, 2009 ▸). Many important proteins, and especially membrane proteins, are thus still purified from native sources. Macromolecular complexes purified from *ex vivo* sources (for example, tissues or blood) sometimes co-purify with endogenous components that are only identified during structure determination (Vallese *et al.*, 2022 ▸), providing fresh insight into their cellular context. Subcomplexes or alternative subunit compositions also coexist, illuminating function, mechanism and native oligomeric states.

*Drosophila* represents a versatile alternative to either native tissue or primary cell lines, which can be challenging to maintain. Not all human proteins have known counterparts in *Drosophila*, but in many cases endogenous complexes can be purified and fusion tags or mutations inserted into a transgene whilst silencing the endogenous protein. Although we did not pursue this further, we found evidence of other mitochondrial proteins, including Cox-II from the inner mitochondrial membrane, co-purifying with TOM, indicating the potential for identifying new mitochondrial assemblies by extraction from *Drosophila* membranes.

Using *Drosophila* as an expression system for structural biology, whilst not trivial, can be variously adapted to improve yields and study eukaryotic complexes produced *in vivo*. While Tom40 expression in photoreceptor neurons, which contain densely packed mitochondria (Eakin, 1972 ▸), was superior to ubiquitous expression using a tubulin promotor, it only represented a tenfold increase over endogenous Tom40 levels (Periasamy *et al.*, 2022 ▸). Co-expression with Myc, which increases cellular mitochondrial volume (Mitchell *et al.*, 2015 ▸; Li *et al.*, 2005 ▸), resulted in yields of TOM that were adequate for structure determination. Thus, the versatility of *Drosophila* genetics enabled the enhanced protein yields needed for determining TOM structure *ex vivo*.

In summary, this study showcases an innovative and viable strategy for the expression of membrane-embedded macromolecular complexes, offering complementary benefits of facilitating structural studies while enabling parallel physiological investigations in a genetically tractable multicellular model organism. Our approach could prove a useful addition to the repertoire of structural biologists studying specific questions or disease models. As advances in cryoEM methods mean that structure determination can now be achieved with submilligram amounts of high-quality protein, *Drosophila* expression may find increasing appeal as a model system for correlating structure with the phenotypic effects of compromised function associated with disease. In particular, it has application in studying processes such as cellular quality-control pathways, cell death and neurodegeneration, which are evolutionarily conserved between *Drosophila* and humans.

## Supplementary Material

PDB reference: *D. melanogaster* TOM core complex, 9etm

EMDB reference: *D. melanogaster* TOM core complex, EMD-19944

Supplementary figures and tables. DOI: 10.1107/S2052252524011011/fq5025sup1.pdf

## Data Availability

This study did not generate unique reagents or code. The cryoEM map and atomic model of the TOM core complex of *D. melanogaster* have been deposited in the Protein Data Bank and in the Electron Microscopy Data Bank with accession codes 9etm and EMD-19944, respectively. No custom code was used to analyse these data and all methods and packages used are cited in Section 2. Further information and requests for resources and reagents should be directed to Jacqueline M. Gulbis (jgulbis@wehi.edu.au), Werner Kühlbrandt (werner.kuehlbrandt@biophys.mpg.de) or Leonie M. Quinn (leonie.quinn@anu.edu.au).
