# Supplementary material for: Structure of an ex vivoDrosophila TOM complex determined by single-particle cryoEM
Source: IUCrJ. 2025 Jan 1;12(Pt 1):49–61. doi: 10.1107/S2052252524011011 (PMC11707698; doi:10.1107/S2052252524011011)
Supplement: Supplementary file 1 [file m-12-00049-sup1.pdf]

IUCrJ

Volume 12 (2025)

Supporting information for article:

Structure of an *ex vivo Drosophila* TOM complex determined by single-particle cryoEM

Agalya Periasamy, Pamela Ornelas, Thomas Bausewein, Naomi Mitchell, Jiamin Zhao, Leonie M. Quinn, Werner Kuehlbrandt and Jacqueline M. Gulbis

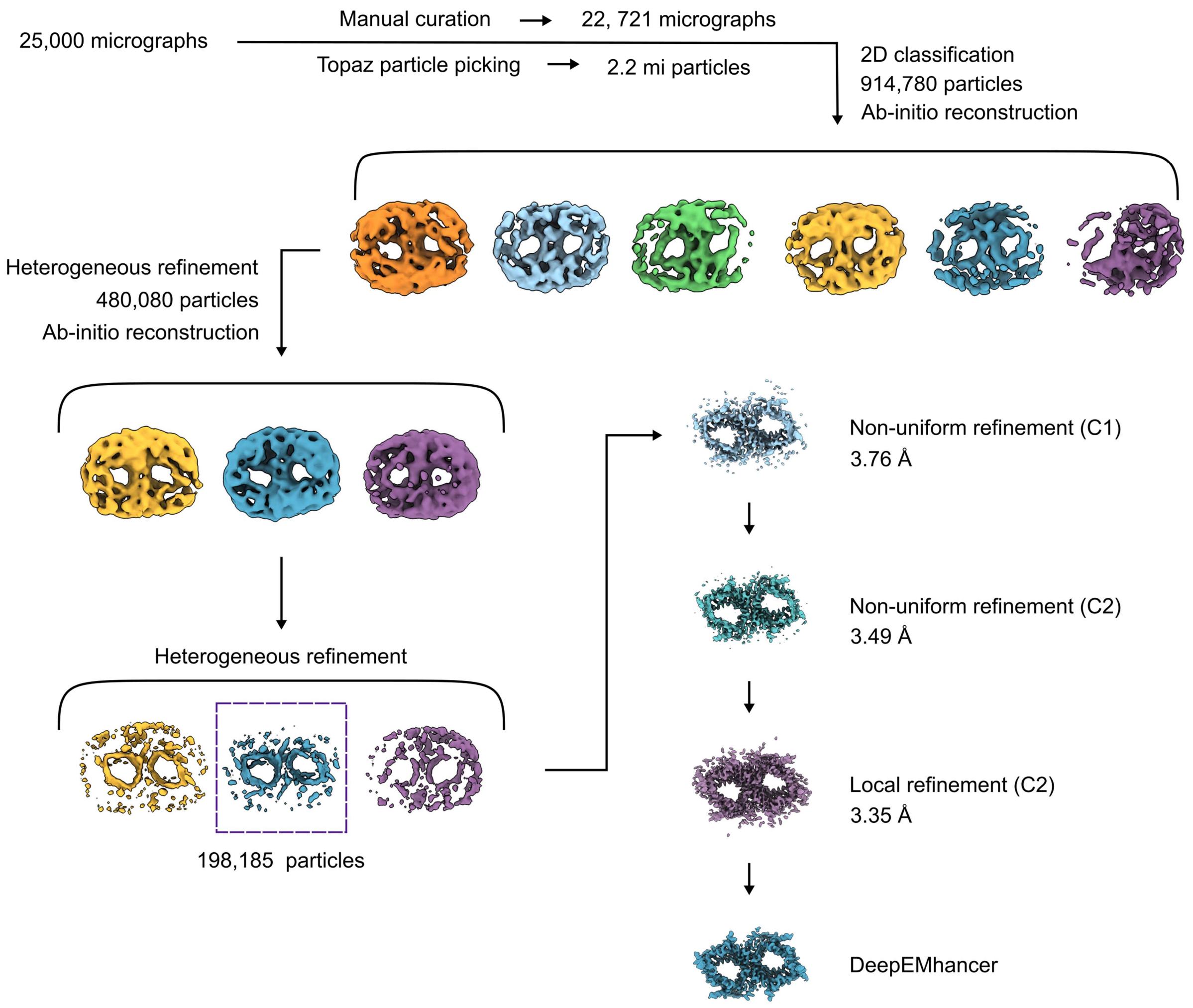

Figure S1 Single-particle analysis of the *Drosophila* TOM core complex. Pipeline leading to the structure obtained in cryoSPARC. The final map reconstruction reached 3.35 Å resolution and was sharpened using DeepEMhancer.

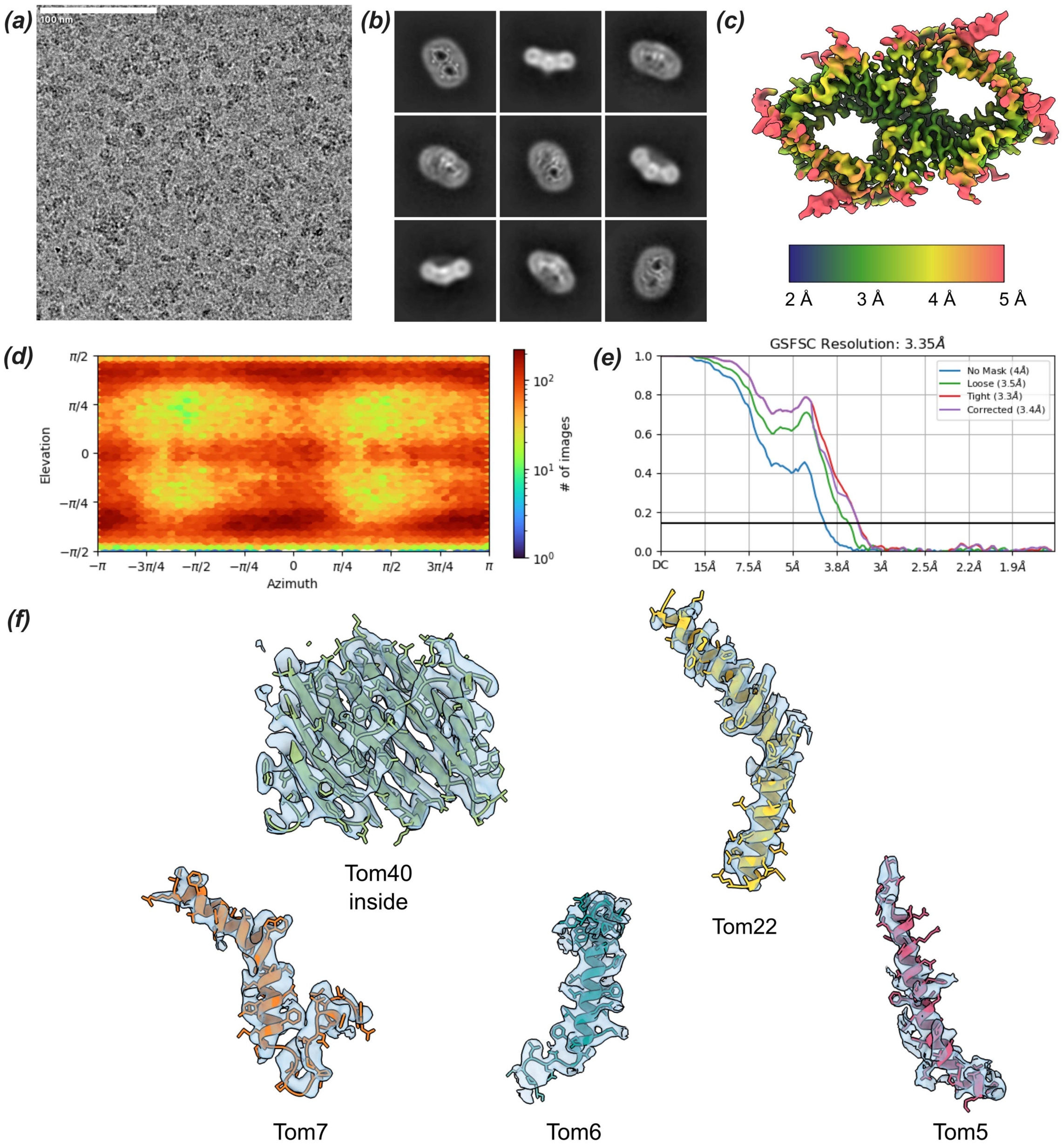

Figure S2 Single-particle processing of *Drosophila* TOM core complex. *(a)* Representative micrograph of graphene back-coated grid. *(b)* Representative 2D class averages. *(c)* Final refined map colored according to local resolution as estimated by cryoSPARC. *(d)* Particle distribution in the final reconstruction presented as a heat map as measured in cryoSPARC. *(e)* Fourier shell correlation of final local refinement and local resolution estimation carried out in cryoSPARC. *(f)* Individual map and model overlays of the subunits of the TOM core complex.

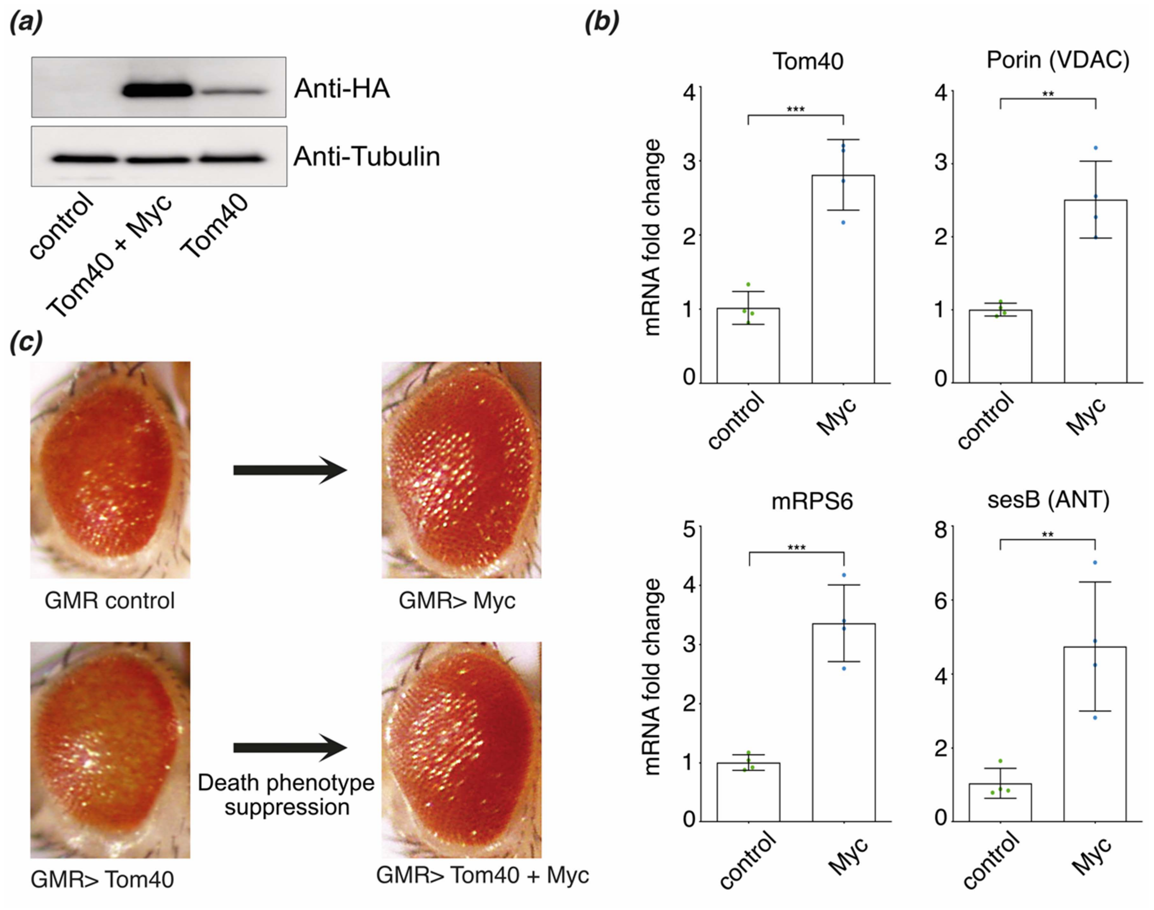

Figure S3 Tom40 expression in *Drosophila* eyes. *(a)* Western blot analysis using anti-HA antibody for Tom40-FLAG-HA co-expressed with Myc. *(b)* qPCR showing mRNA abundance of endogenous mitochondrial genes, including *Tom40*, *Porin*, *mRPS6* and *sesB*, for Myc overexpressing larval heads compared with driver alone control. Myc expression was induced ubiquitously using tubulin-GAL4 and temporally for 48 hours using a temperature-sensitive gal80. In each panel, data from at least three independent biological replicates are represented as mean ± SD. Asterisks indicate p-values ≤0.05 by an unpaired T-test. *(c)* Representative eye images for GMR-driven expression of Myc (GMR-GAL4; UAS Myc) without (above) or with (below) co-expression of Tom40 (GMR-GAL4-Tom40/+; +/UAS Myc).

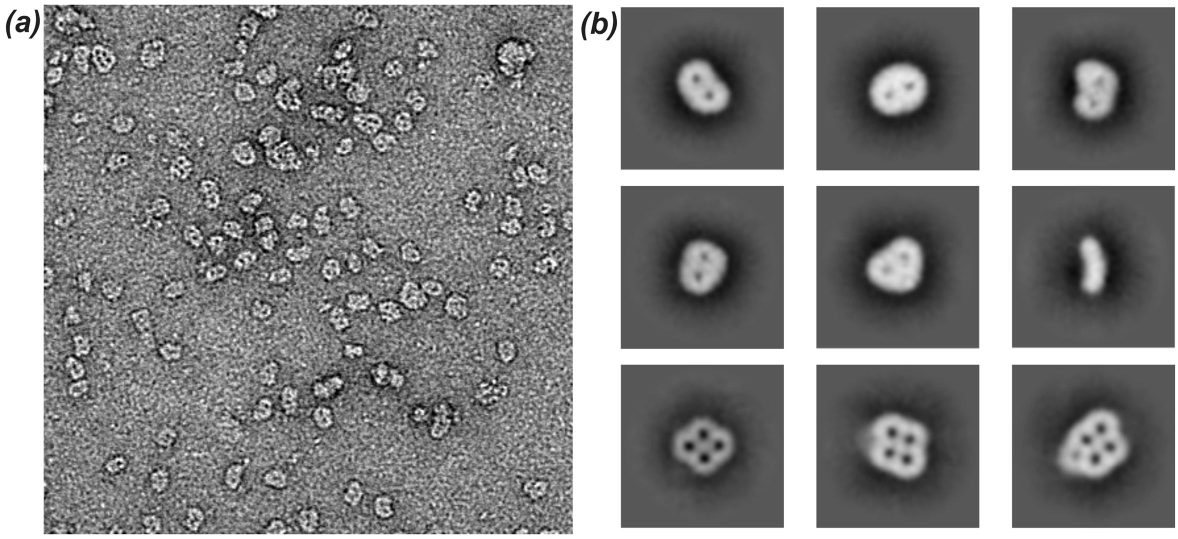

Figure S4 Preliminary negative-stain analysis of TOM complex extracted with TetraDM/CHS and reconstituted into NAPol. *(a)* Close-up of a representative micrograph showing TOM particles with varying pore numbers. *(b)* Representative 2D class averages from classification of 22,000 particles.

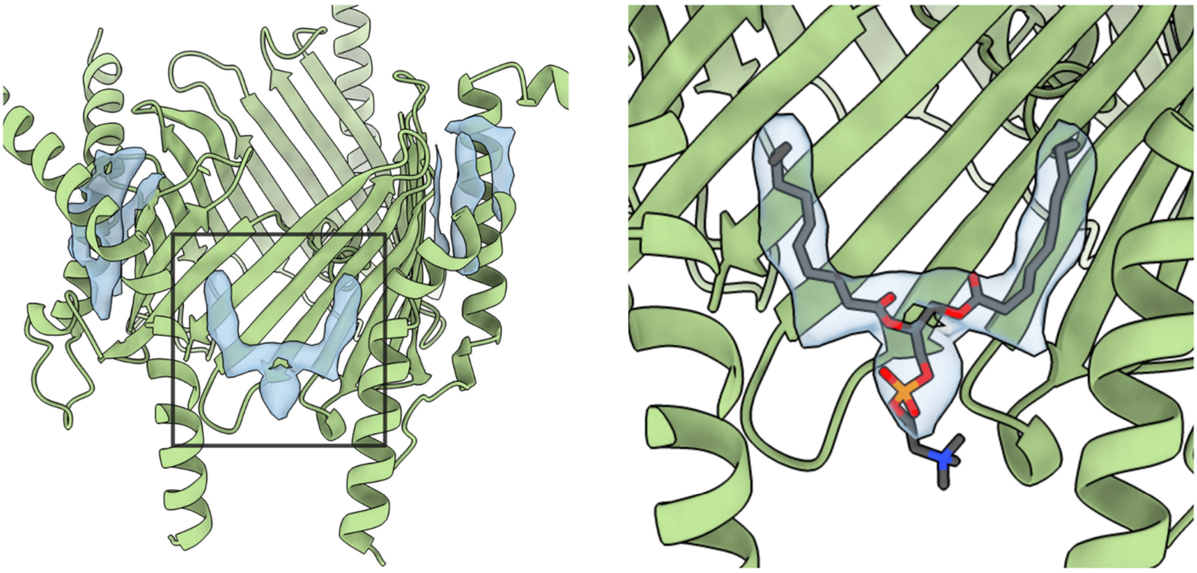

Figure S5 Modelling of a central lipid at the dimer interface. Side view of the *Drosophila* TOM model, cut in between the two Tom40 barrels. The overlay of the lipid regions in the symmetry-imposed map (blue) were modelled as phosphatidylcholine.

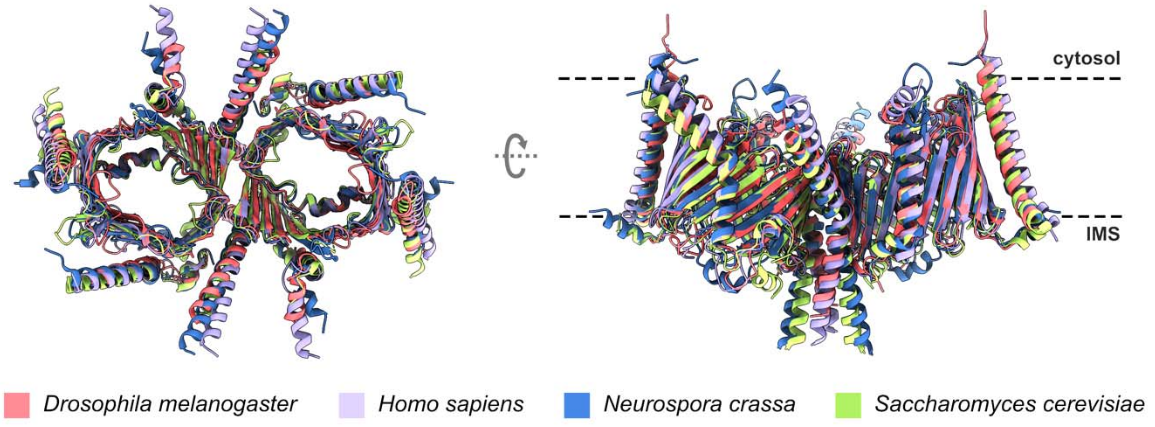

Figure S6 Comparison of *Dm*TOM to previously published TOM models. Overlay of structures of TOM from *Drosophila melanogaster* (red; PDB 9ETM); *Homo sapiens* (violet; PDB 7CK6), *Saccharomyces cerevisiae* (green; PDB 6UCU) and *Neurospora crassa* (blue; PDB 8B4I).

```
H.sapiens           LALGAFLNHRKNKFQCGFGLTIG  361
M.musculus          LSLCAFLNHRKNKFLCGFGLTIG  361
X.tropicalis        LAMGAFLNHKKNKFQCGFGLTIG  336
D.rerio             LALGAFLNHRKNKFQCGFGVTIG  360
D.melanogaster      LALSGRMNHVKNNFRLGCGLMIG  344
C.elegans           LALSGTLNHVKAAGKFGIGLIIG  301
                    *:: . :** *       *: **
```

Figure S7 Sequence alignment of the Tom40 C-terminal region. Tom40 sequences from higher eukaryotes of the animal kingdom were aligned using Clustal Omega. Primary accession numbers of *Homo sapiens*, *Mus musculus*, *Xenopus tropicalis*, *Danio rerio*, *Drosophila melanogaster* and *Caenorhabditis elegans* used were O96008, Q9QYA2, Q6P825, A0A8M6Z1R1, Q9U4L6 and Q18090, respectively.

(a)

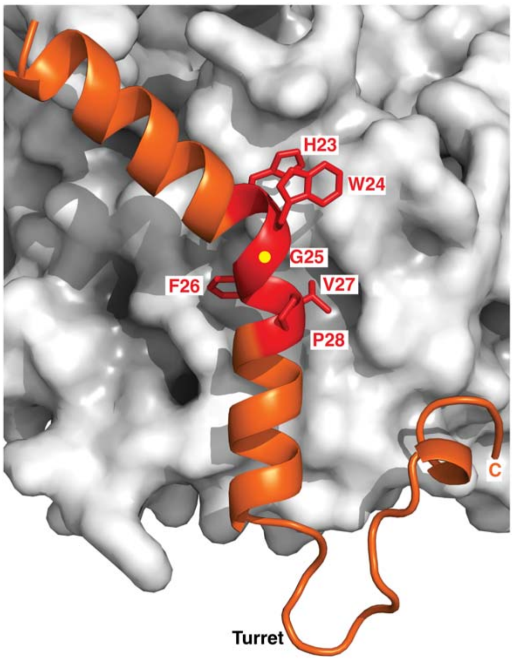

(b)

```
D. melanogaster -MKLSEGVKDRLGFVVGVVQTGFHWGFVPLVLYLGFMKGAEPGMPPLNLFSLLWQ 54
     H. sapiens MVKLSKEAKQRLQQLFKGSQFAIRWGFIPLVIYLGFKRGADPGMPEPTVLSLLWG 55
                 :***: .*:**  :.   * .::***:***:**** :**:****  .::****
```

Figure S8 The GxxP motif and surrounding residues of Tom7. (*a*) Ribbon depiction of Tom7 at the Tom7-Tom40 interface in the *Drosophila* TOM complex. Tom40 is shown as a solvent accessible surface (grey). Side chains of the amino acid residues near the conserved GxxP sequence are shown as stick models. The position of the glycine residue is indicated by a yellow filled circle. The F26 and V27 residues of GxxP occupy surface pockets of Tom40. (*b*) Sequence alignment of human and *Drosophila* Tom7, showing conservation of the motif, with a red bar indicating the depicted sequence.

Table S1 List of fly strains used in the study.

D. melanogaster: UAS-Tom40-FLAG.HA	DPiM: 0631	Guruharsha, K.G., Obar, R.A., Mintseris, J., Aishwarya, K., Krishnan, R.T., VijayRaghavan, K., Artavanis-Tsakonas, S., 2012. Drosophila Protein interaction Map (DPiM) A paradigm for metazoan protein complex interactions. Fly (Austin) 6, 246–253.
D. melanogaster: GMR-GAL4<br>w1118; P{GMR-GAL4.w-}2/CyO	BDSC: 9146	Perrin, L., Bloyer, S., Ferraz, C., Agrawal, N., Sinha, P., Dura, J.M. (2003). The leucine zipper motif of the Drosophila AF10 homologue can inhibit PRE-mediated repression: implications for leukemogenic activity of human MLL-AF10 fusions. Mol. Cell. Biol. 23(1): 119--130.
D. melanogaster: tubulin-GAL80ts<br>w[*]; P{w[+mC]=tubP-GAL80[ts]}20; TM2/TM6B, Tb[1]	BDSC: 7019	McGuire, S.E., Le, P.T., Osborn, A.J., Matsumoto, K., Davis, R.L. (2003). Spatiotemporal rescue of memory dysfunction in Drosophila. Science 302(5651): 1765--1768.
D. melanogaster: w1118	BDSC: 3605	Hazelrigg, T., Levis, R., Rubin, G.M. (1984). Transformation of white locus DNA in Drosophila: Dosage compensation, zeste interaction, and position effects. Cell 36(): 469--481.
D. melanogaster: UAS-Myc<br>w1118; P{w[+mC]=UAS-Myc.Z}132	BDSC: 9674	Shcherbata, H.R., Althauser, C., Findley, S.D., Ruohola-Baker, H. (2004). The mitotic-to-endocycle switch in Drosophila follicle cells is executed by Notch-dependent regulation of G1/S, G2/M and M/G1 cell-cycle transitions. Development 131(13): 3169--3181.

Table S2 CryoEM data collection, refinement and validation of the DmTOM core complex.

	TOM core
**Data collection and processing**	
Magnification	105kx
Voltage (kV)	300
Electron exposure	55 e-/A2
Defocus Range (μm)	-1.4 to -2.4
Pixel size (Å)	0.837
Symmetry imposed	C2
Initial particles	914,000
Final particles	198,185
Map resolution (Å)	3.35
FSC Threshold	0.143
**Refinement**	
Initial model used	AlphaFold-Multimer
Model resolution	3.34
FSC Threshold	0.143
Map sharpening B factor ($Å^2$)	114
**Model composition**	
Nonhydrogen atoms	7404
Protein residues	928
Ligands	5
**R. m. s. deviations**	
Bond lengths (Å)	0.003
Bond angles ()	0.642
**Validation**	
MolProbity score	1.91
Clashscore	2.08
Poor rotamers (%)	0.00
Ramachandran plot	
Favored (%)	97.25
Allowed (%)	2.75
Disallowed (%)	0.00

Table S3 Percentage of similarity/identity of core TOM components between different species.

Species	% Identity/Similarity to human orthologs		
	Tom40	Tom22	Tom7
Drosophila	51.8/65.5	38.3/54.5	49.1/63.6
Saccharomyces	23.5/40.1	18.2/32.4	25.0/45.3
Neurospora	22.6/39.3	20.6/37.5	33.3/63.2
